# The systemic effects of 22q11.2 deletion syndrome on immunity

**DOI:** 10.70962/jhi.20250190

**Published:** 2025-10-30

**Authors:** Nicolai S.C. van Oers, Kathleen E. Sullivan

**Affiliations:** 1 Departments of Immunology, Microbiology and Pediatrics, The University of Texas Southwestern Medical Center, Dallas, TX, USA; 2 Division of Allergy and Immunology, Children’s Hospital of Philadelphia, Philadelphia, PA, USA

## Abstract

22q11.2 deletion syndrome (22q11.2DS) affects about 1/2,150 individuals, causing complex and variably penetrant clinical problems. The clinical phenotypes evident at birth can include thymic hypoplasia, hypoparathyroidism, heart defects, and/or facial dysmorphism. Neurological issues including behavioral problems such as autism spectrum disorders and schizophrenia are evident at later postnatal periods. Thymic hypoplasia affects about 60–70% of patients, leading to T cell lymphopenias of varying severity. In rare cases, a congenital athymia occurs, necessitating a thymic implant. This review provides information regarding the causes and consequences of 22q11.2DS on thymic functions along with its broader impacts on the immune system. The affected immune cells include T, B, and mast cells. Patients with 22q11.2DS have more infectious, autoimmune, and allergic complications. Broader systemic changes including increased vascular permeability, a disrupted blood–brain barrier, and epigenetic alterations resulting from deletions on chromosome 22q11.2 affect many organ systems that can involve immune responses.

## Teaser

22q11.2 deletion syndrome has complex clinical presentations, with congenital malformations of the thymus leading to many immune system changes. These are summarized in the current review.

## Introduction

### Overview

**Figure 1.**
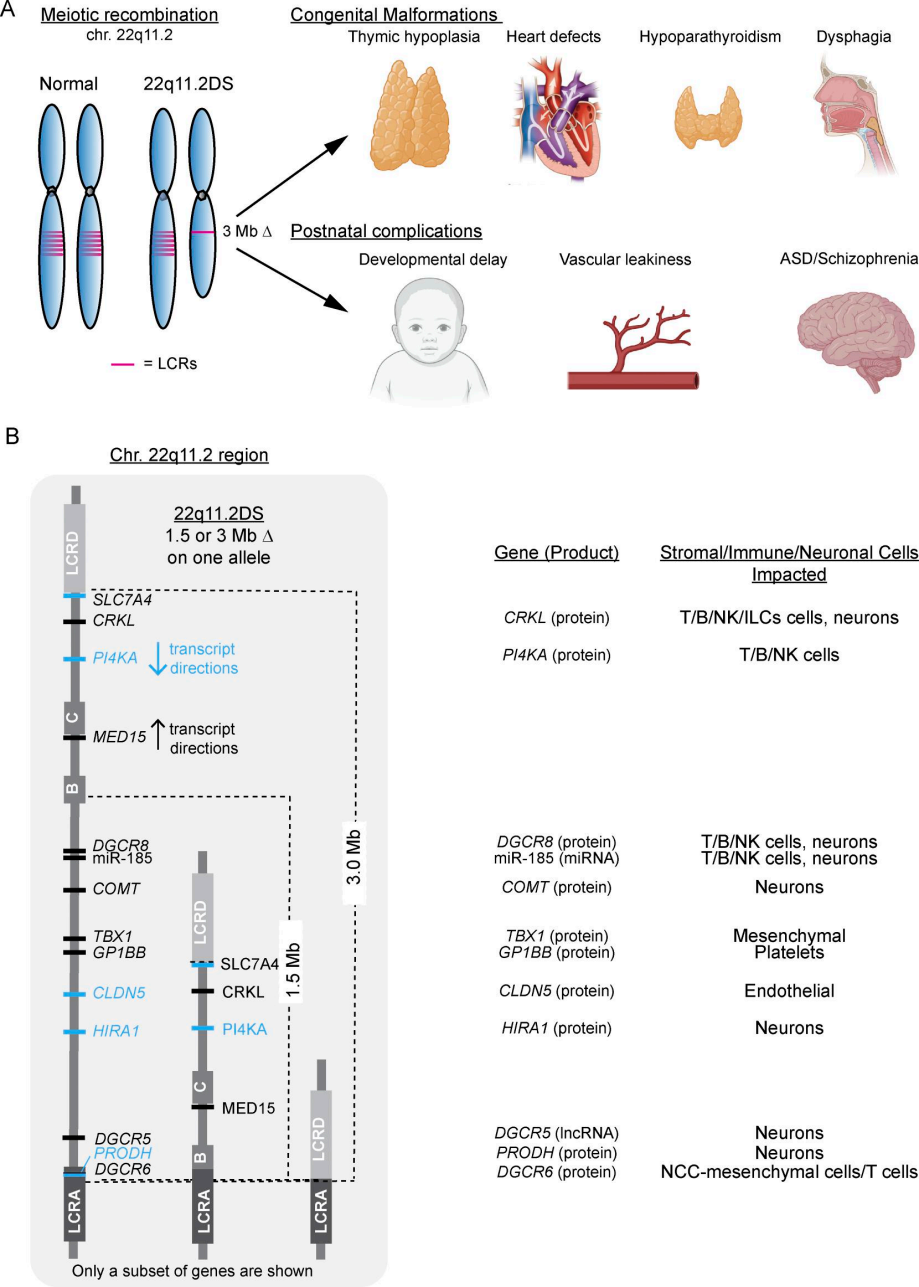
**Clinical phenotypes associated with genes on the frequently deleted segments of chr. 22q11.2. (A)** Fetal and postnatal clinical problems associated with 22q11.2DS arising from chromosomal deletions on 22q11.2. The red lines depict several of the low copy repeats (LCRs) in the chr. 22q11.2 region that result in the 3- and 1.5-Mb deletions. **(B)** Chromosomal map with several of the genes indicated along with their impact on the immune and neuronal systems. The blue and black defines the transcript direction. Note that *PRODH* and *DGCR6* are encoded at the same location and transcribed in opposite directions. BioRender was used for components of the image. ILC, Innate lymphoid cells.

22q11.2 deletion syndrome (22q11.2DS, a.k.a. DiGeorge syndrome) is a multi-syndromic condition consisting of congenital malformations arising during embryogenesis along with later onset neurological complications (1, 2, 3, 4, 5, 6, 7). The prevalence of 22q11.2DS is 1/2,150 live births (2). The extent and severity of the congenital problems due to 22q11.2DS are highly variable and can include thymic hypoplasia, hypoparathyroidism, cardiac outgrowth vessel defects, and/or dysmorphic facial features (Fig. 1 A) (1, 3, 4, 6, 8, 9, 10, 11, 12). Postnatal issues for patients encompass developmental delay, epilepsy, autism spectrum disorder, and/or schizophrenia (Fig. 1 A) (13, 14, 15). These diverse and broad clinical symptoms result from a common 3 Mb or less frequent, nested 1.5 Mb deletion on chromosome 22q11.2 (chr. 22q11.2), which arises because of improper meiotic recombination (Fig. 1 B) (16, 17). Duplication of chr. 22q11.2 results in some overlapping phenotypes as the deletions, although these are less severe and less penetrant (Table 1) (18). In 22q11.2DS, a recurrent 3-Mb deletion occurs in ∼85–90% of individuals, creating a haploinsufficiency of about 146 genes. These comprise 46 coding and the remainder noncoding elements (e.g., microRNAs [miRNAs], long noncoding RNAs [lncRNAs], and other small non-coding RNAs [sncRNAs] (Fig. 1 B and Table 1) (4). Among the 46 coding genes, a haploinsufficiency of *T-box transcription factor 1* (*TBX1*) is the principal driver of the embryonic malformations. This was confirmed in patients carrying just *TBX1* variants (loss- and gain-of-function consequences), whose clinical presentations can include thymic hypoplasia, hypoparathyroidism, aortic arch defects, and/or dysmorphic facial characteristics (Table 1) (19, 20). While a haplosufficiency of *TBX1* drives the congenital problems, the remaining coding and noncoding genes in the deleted region do influence disease severity (4). The clinical impact on the immune system depends primarily on whether the thymus is hypoplastic, severely hypoplastic, or aplastic (Table 2). The consequent medical care needs for 22q11.2DS patients are complex, and costs can exceed one million dollars during an individual’s first two decades of life (21, 22, 23, 24, 25, 26, 27). In this review, we will address pathophysiologic mechanisms, clinical findings, and important questions still outstanding regarding 22q11.2DS.

**Table 1.** Clinical presentations in 22q11.2DS compared to duplications on 22q11.2 and *TBX1* variants

Clinical disorder	22q11.2DS LCRA-D 3-Mb deletion 22q11.2DS LCRA-B 1.5-Mb deletion	22q11.2DS duplication	TBX1 loss or gain of function
Frequency	1/2,150	1/1,600	Rare (∼10–15 families)
Congenital problems: % affected			
Thymic hypoplasia	70–80%	1–2%^a^	20–45%
Thymic aplasia	<1%	Not reported	Not reported
Hypocalcemia	50–65%	2.8%	45%
Cardiac defects	60–85%	4–6%	56%
Craniofacial features	80–90%	12.7%	100%
Speech delay (GERD^b^)	70–90%	46%	33%
Postnatal concerns: % affected			
Immunological			
Allergy	60%	25%	Unknown
Autoimmunity	5–10%	2.3%	Unknown
CD3^+^ T cell lymphopenia	60–70%	2.6%	Unknown
Decreased class switch memory B cells	70%	25%	Unknown
Low pneumococcal vaccine responses	39%	0%	Unknown
Hypogammaglobulinemia	6%	10–20%	Unknown
Neurological			
Developmental delay	8–10%	45–50%	30%
Autism spectrum disorder	15–40%	20%	38%^c^
Schizophrenia	30–35%	Protective	38%^c^
Disrupted blood–brain barrier	Unknown	Not reported	Not reported

a Reflects low peripheral T cells.

b Gastroesophageal reflux disease.

c Listed as combined psychiatric disorders.

**Table 2.** Clinical range of T cell lymphopenias among 22q11.2DS patients

Thymic hypoplasia (60–70%)	More severe thymic hypoplasia	Congenital athymia (<1%)	Normal thymus (30–35%)
Reduced CD3^+^ T cells (<1,500 cells/µl)	Low CD3^+^ T cells (>50 and <400 cells/µl)	Very low CD3^+^ T cells (<50 cells/µl)	CD3^+^ T cells >1,500 cells/µl
Reduced numbers of naïve T cells and expanded memory T cells	Low naïve T cells (>50 cells/µl; >5% and <50% total T cells)	Low naïve T cells (<50 cells/µl; <5% total T cells)	Higer naïve to memory T cell ratios (infants)
TREC levels are not flagged as abnormal	Undetectable or very low TRECs^a^	Undetectable or very low TRECs^a^	Normal TRECs
Elevated T follicular helper cells	​	Possible maternal engraftment	​
Treatment strategies			
Monitor and possible prophylactics	Monitor and possible prophylactics	Thymic implant (transplant)	Not applicable

a Flagged by newborn screening and may vary between testing centers.

### Diagnosis of thymic hypoplasia

Given the multiple congenital problems associated with 22q11.2DS, early fetal screening is beneficial to identify those who may need advanced clinical care at birth. One early screen, starting at week 10 of gestation, involves next-generation sequencing of cell-free DNA obtained from maternal plasma (28). Diverse microdeletions, such as 22q11.2DS, can be detected in fetal DNA from the plasma (29). This microdeletion screen may be recommended if parents are known carriers of 22q11.2DS and/or there are suggestive prenatal indicators (30).

Congenital heart abnormalities are the most common birth defect in humans, and 60–80% of 22q11.2DS patients have cardiac anomalies (8, 30). Standard of care prenatal ultrasound examinations includes fetal heart monitoring to detect cardiac anomalies (31). High-resolution 2D fetal echocardiography, often undertaken to characterize the cardiac problems, can also reveal a small thymus. This is done by measuring the ratio between the diameter of the thymus (T) (anteroposterior measure) and the intrathoracic (T) mediastinum (11, 32). A normal human fetal thymus has a mean T/T ratio of 0.44, while 22q11.2DS fetuses have a mean value of 0.25 (11, 32). A reduced fetal T/T ratio is suggestive of thymic hypoplasia, which correlates with lower T cell counts postnatally (33). Not surprisingly, lower T cell receptor excision circles (TRECs) are also associated with lower naïve CD4 T cell counts (34).

A small fraction of 22q11.2DS patients can be picked up through newborn screening using a PCR screen that detects TRECs (35, 36, 37, 38). TRECs form as T cells rearrange the TCR-α locus in the thymus, and these excised DNA circles remain in the recent thymic emigrants (39). Low-to-absent TREC values indicate low T cells, and 22q11.2DS is one of the more common conditions identified in this newborn screen (35, 40, 41). A repeat low-to-undetectable TREC result suggests an inborn error of immunity (IEI) (14, 42, 43). To confirm if a patient has deletions on chr. 22q11.2 versus distinct genetic mutations causing SCID, a chromosomal microarray analysis, multiplex ligation–dependent probe amplification, or alternative copy number analysis is required. It is estimated that <1% of patients with 22q11.2DS are identified through newborn screening (44). This is because 60–70% of 22q11.2DS patients have low but sufficient naïve T cells, with TREC levels not flagged by newborn screens (Table 2). Less than 1% of 22q11.2DS patients have congenital athymia, necessitating a thymic implant (referred to as a transplant in the UK) (Tables 1 and 2) (14, 25, 40). Congenital athymia in 22q11.2DS patients results from thymic stromal cell defects, leading to a T^^−^^B^+^NK^+^ phenotype with an absence or severe reduction in naïve T cells (14, 40). Patients with thymic aplasia have absent TRECs, failing newborn screens for those countries that offer newborn screening for SCID. Many studies have provided clinical practice guidelines for 22q11.2DS patients (Table 2) (1, 6, 9, 14, 25, 45).

### Thymic tissue development in 22q11.2DS

The thymic anlage and inferior parathyroids form within the third pharyngeal pouch (third PP) during embryogenesis (weeks 7–8 of gestation in humans, days embryonic (E) E10.5–E11.5 in murine embryos) (46). The thymic hypoplasia/aplasia in the 22q11.2DS cohort begins at this early development stage. Confirming this, thymic hypoplasia is already present in murine embryos isolated between E11 and E13 from various mouse models of 22q11.2DS (47, 48, 49, 50, 51, 52, 53). In such embryos, the third PP and third and fourth pharyngeal arch arteries are often underdeveloped or absent (47, 48, 49, 50, 51, 52, 53). While developmental problems of the third PP explain the thymic and parathyroid changes, alterations in the third and fourth pharyngeal arches lead to abnormal patterning of the aortic arch, along with the carotid and subclavian arteries. This explains the outflow track problems in 22q11.2DS patients (8). Murine embryos and postnatal mice also exhibit dysphagia, revealing the extensive overlapping congenital abnormalities between human 22q11.2DS and mouse models (52, 54).

**Figure 2.**
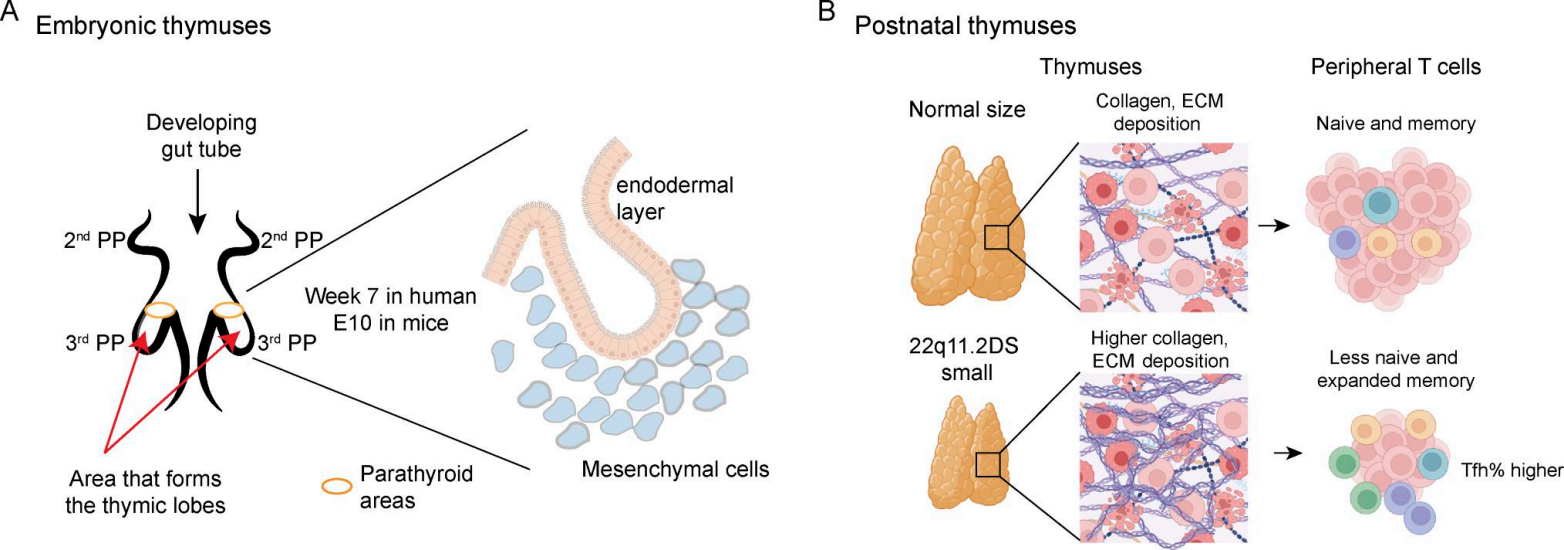
**Thymus specification and expansion during embryogenesis in normal and 22q11.2 settings. (A)** The thymus and inferior parathyroids are formed from the third PP, a temporary evagination of the gut tube during embryogenesis. The tissue is specified as NCC–derived mesenchymal cells surrounding the endothelial layer and promoting the 3D thymic structure. **(B)** The thymus in 22q11.2DS is smaller than normal controls, with increased collagen deposition due to an expanded chondrocyte population. There are fewer naïve T cells in 22q11.2DS, with evidence of an expansion of memory cells, including an elevated percentage of Tfh. Biorender was used for a portion of the image.

The ability of thymic implants (transplants) to reconstitute T cell development in 22q11.2DS patients with congenital athymia suggests the molecular cause of thymic aplasia is a stromal cell problem. This is because the donor thymus is cultured for 3 weeks prior to implant, with most thymocytes dying (24, 55, 56). The remaining thymus is primarily composed of stromal cells; thymic epithelial cells (TECs), fibroblasts, and the endothelial vasculature (57). Implanted into the quadriceps, the donor thymus attracts host hematopoietic cells that differentiate into thymocytes (24, 25, 55). A candidate stromal cell hypothesized as causal to 22q11.2DS phenotypes was the neural crest cell (NCC)–derived mesenchymal cell (58, 59). During the formation of the thymus, such NCC-derived mesenchymal cells surround the single endothelial layer within the third PP (Fig. 2 A) (46, 60). The mesenchymal cells induce endothelial cell-to-TEC transitions. Subsequent multicellular interactions between the mesenchymal cells, expanding TECs, and early immature CD4^−^CD8^−^ thymocytes create a 3D spongelike meshwork of cells unique to the thymus (61, 62, 63). Studies comparing the developing embryonic thymuses from normal and 22q11.2DS murine models established key alterations in the mesenchymal cell subsets (64). In the Tbx1^neo2/neo2^ mouse model (36% normal levels of Tbx1), embryos from E12 to E17.5 had a penetrant thymic hypoplasia, an interrupted aortic arch, and mispositioned parathyroids (49, 65, 66). Single-cell RNA sequencing of normal embryonic thymuses from E13 to E13.5 gestational days revealed six, five, and four mesenchymal, TEC, and hematopoietic cell subsets, respectively, along with one endothelial population (64, 67, 68). Mesenchymal cells from the hypoplastic thymuses (Tbx1^neo2/neo2^ embryos) had an altered trajectory with pronounced expansion of chondrocytes (67). Chondrocytes are major producers of collagens and extracellular matrix (ECM) proteins, which can increase tissue stiffness, reduce tissue expansion, and impair vascular development (69, 70, 71). Immunofluorescence studies confirmed elevated collagens and ECM proteins in both murine embryonic thymuses and postnatal thymuses from human 22q11.2DS patients (72). Of note, replacing the Tbx1^neo2/neo2^ mesenchymal cells with normal ones in the murine embryos restored embryonic thymic tissue growth in reaggregate thymic organ culture assays (64). Single-cell RNA sequencing confirmed that the murine “22q11.2” thymic mesenchymal subsets had altered transcriptomes and trajectories, while TECs had minimal alterations (67, 73). A significant advance was the discovery that administration of either minoxidil or PGE_2_ in the pregnant mouse models of 22q11.2DS normalized thymic tissue growth (67). These drugs prevented chondrocyte expansion and limited collagen and ECM production. Interestingly, while most of the principal transcripts involved in TEC functions were normal, several Sox-family transcription factors (TFs) were elevated in the developing immature TECs in the mouse embryos (72). It is not known how this might influence the composition of TEC mimetics and medullary TEC subsets that have developed tissue/organ specific identities by upregulating lineage defining TFs (74). Single-cell RNA sequencing of postnatal human thymuses from 22q11.2DS patients was recently reported, further supporting a mesenchymal cause for thymic hypoplasia (unpublished data). Comparisons with non-22q thymuses revealed that the 22q11.2DS tissues had altered biological pathways, with top hits including ECM assembly and structure, collagen production, and fibril organization, along with vascular and connective tissue development. The human 22q11.2DS thymuses had diminished medullary regions, with more collagen deposition throughout the thymus.

### T cell development in 22q11.2DS thymuses

In a normal thymus, T cell development follows an ordered progression of immature CD4^−^CD8^−^ (double negative [DN]) to CD4^+^CD8^+^ (double positive [DP]) followed by CD4^+^CD8^−^ and CD4^−^CD8^+^ single positive (SP) thymocyte subsets (75, 76). Comparative analyses of human thymuses from 22q11.2DS (thymic hypoplasia) and non-22q patients have revealed similar processes of T cell development (77). However, the overall size and cellularity of the thymuses were lower than controls (64, 77, 78). The percentages of DN, DP, and SP subsets remained similar, implying normal TEC functions (64, 77, 78). An initial report suggested that thymic T regulatory cells (Tregs) from 22q11.2DS patients had diminished suppressive activities; this was not observed in other findings (77, 78). Taken together, the data concur that 22q11.2DS leads to a smaller thymus that exhibits relatively normal thymopoiesis (53). Recent spatial transcriptomic and cellular indexing of transciptomes and epitopes (CITE)-Seq have yielded numerous detailed processes governing T cell development in the thymus (79). This includes the identification of more than 50 different cell subsets, including 15 TEC mimetics. A thorough comparison of these diverse cell types in human 22q11.2DS thymuses will reveal how the mesenchymal cell changes influence the other stromal and hematopoietic cell subsets.

### The impact of 22q11.2DS on peripheral T cell subsets

The classic finding in 22q11.2DS patients with low T cell numbers is a reduction in their naïve CD4 T cells (Table 2) (34, 80). Multiple studies have examined the impact of 22q11.2DS on specific T cell subsets among these CD4 T cells. There are more memory T cells at all ages, with concordant increases among the Th1, Th2, and Th17 helper T cell subsets (34). Th17 cells were highest in those children who had the lowest TRECs (detected at birth). These Th17 cells also had chromatin signatures of senescence and inflammation (also documented by flow cytometry) (81, 82, 83). It remains unclear as to whether an inflammatory milieu led to this senescence phenotype (84). Other T cell types impacted by 22q11.2DS are less clearly linked to thymic hypoplasia. For example, follicular helper T cells (Tfh) actually increased in the 22q11.2DS cohort compared to normal controls (85). This contrasts with the decreased number of Tregs (86, 87), although Treg differences are not consistently observed (78, 88). Such T cell findings are difficult to align with just a simple thymic hypoplasia and may reflect compromised thymic structure or vasculature (72, 77). Immaturity in the structure of the thymus has been observed with concomitant decreased suppressor function of regulatory T cells, which is not always noted (77, 78). This and/or the reduced size of the medullary region of the thymus, potentially impacting TEC mimetics, could be contributors to increased autoimmune diseases. Increased Tfh cells may be both a reflection of autoimmune disease and differential gene expression (89, 90).

### Clinical features

Most 22q11.2DS patients have reduced numbers of circulating T cells relative to age-matched cohorts due to thymic hypoplasia (Table 2) (1, 3). Thymic aplasia (congenital athymia) is rare (<1% of 22q11.2DS patients). For congenital athymia, cultured thymic implants have been performed for decades and, at present, are primarily available in the U.S. and Great Britain. Referral to a center for thymic implantation is based on laboratory features demonstrating low TRECs, low-to-absent naïve T cells, and poor T cell mitogen responses (14). For those on the more extreme degree of T cell lymphopenia but not eligible for a thymic implant (>50 to <400 CD3^+^ cells/µl), clinical considerations should include prophylactic treatments and immunoglobulin therapies (14, 40). Unfortunately, there are no therapeutic strategies to increase circulating T cell numbers for 22q11.2DS patients with persistent T cell lymphopenia. Another important consideration is whether the 22q11.2DS patient may have had a partial or total thymectomy because of their cardiac surgery (91, 92, 93). T cell lymphopenia occurs after thymectomy in all patients (reviewed in 94). While thymectomies were not considered a clinical problem initially, recent studies have shown that infants/toddlers who had their thymuses removed do have long-term health complications (92). This includes higher mortality rates at younger ages and greater risk scores for both cancer and autoimmunity compared to those whose thymus was retained during cardiac surgery (91, 92, 95, 96). The specific consequences of thymus removal in the 22q11.2DS cohort remain to be defined.

### Low peripheral T cell numbers and infectious risks

Thymic hypoplasia occurs in 60–70% of 22q11.2DS patients (3, 6). A smaller thymus leads to an ensuing peripheral T cell lymphopenia, with impacted individuals having more frequent infections and longer recovery periods compared to age-matched controls (1, 3, 14, 97). Within their first year of life, 22q11.2DS patients have peripheral CD3^+^ T cell numbers with a mean of 1,625 cells/µl (newborns) and 1,823 cells/µl (at 1 year of age) compared to the mean of 1-year-old controls at 3,400 cells/µl (98). This is an overriding basis for immune dysfunction in 22q11.2DS, previously referred to as DiGeorge syndrome in older publications. The peripheral T cell lymphopenia is well-defined in children (98, 99, 100, 101). Adults with 22q11.2DS have T cell counts that are typically in the normal range (102, 103, 104). Yet, adults continue to get infections and higher rates of autoimmunity, supporting the concept that 22q11.2DS has long-term effects on the immune system (99).

Concerning infectious susceptibilities, the first descriptions of infections in those with 22q11.2DS (a.k.a. DiGeorge syndrome) focused on critically ill infants who likely had absent thymic tissue (105, 106). Candida was common along with viral infections that were often fatal (99). Today, such infants would be classified as having congenital athymia and, much like SCID, would be immediately recognized as having a life-threatening condition requiring strict isolation along with rapid therapeutic interventions (Table 2) (14, 35, 41, 42). In one study of genetically heterogeneous babies with athymia, 10/12 had significant infections prior to thymic implantation (55). As has been stressed in this review, most patients with 22q11.2DS have thymic hypoplasia as opposed to aplasia. In a large cohort study of these 22q11.2DS patients, 78% reported recurrent or severe infections including pneumonia, with 27% requiring hospitalization for their infections (107). Thus, an anticipated consequence for the 22q11.2DS cohort with low T cells is infections (108). Most studies have not found a strict association between T cell numbers and infections, although one study found an association of low effector memory CD8 T cells with increased infectious incidents (103, 109). Similarly, TREC counts have not been associated with infections. Intrinsic T cell functions and proliferative responses are usually normal. 22q11.2DS patients do not have typical opportunistic infections as was seen in the original cohorts of HIV infected individuals, who presented with a similar magnitude of T cell lymphopenia. In HIV, T cells are one of the viral targets, which complicates the comparison.

The median number of infections for a 22q11.2DS cohort ranges from 2 to 10/year. Pneumonia, otitis media, and sepsis are the three most common infections, with 83.3% of 22q11.2DS patients having had at least one hospitalization for these infections (110). In another large cohort study specifically focusing on patients with 22q11.2DS, serious infections were reported in 33%. Two patients had opportunistic infections: *Pneumocystis jiroveci* and *Mycobacterium abscessus*; 14% of patients died, 75% of those deaths from infections (103). In a study of adults with 22q11.2DS, infections persisted as a concern, as 38% had recurrent pneumonias, 35% had recurrent otitis media, 11% had recurrent sinusitis, and 35% had seborrhea (111). Other clinical considerations for 22q11.2DS patients include their past medical surgeries and/or additional genetic alterations. For example, those who had cardiac surgeries could have altered right atrial pressures and/or ligation/scarring of the thoracic duct. This could affect immune cell trafficking and circulating cell numbers (112). Some may have had chylothorax as a complication, with increased infections a recognized outcome due to lymphopenia secondary to chylothorax (113). Long-term studies of adults who have had thoracic duct ligation have not demonstrated any increase in infections (114, 115). In children who have impaired thoracic duct flow due to high right atrial pressures, the infections were restricted to warts (116). In summary, aberrant distribution of T cells has a limited impact on infection risk in non-22q11.2DS patients.

### Vaccinations in the 22q11.2DS cohort

In 22q11.2DS patients with congenital athymia, live viral vaccines should not be administered. In the information from vaccine package inserts, it is recommended that live viral vaccines not be given to immunodeficient individuals. However, there is no clear delineation of what constitutes immunodeficiency. The Infectious Disease Society of America (IDSA) and American Academy of Pediatrics (AAP) recommend vaccinations for children with CD8 T cells >200 cells/µl and normal T cell proliferative responses to mitogens (117, 118). Five separate studies of 22q11.2DS patients investigated the safety of live viral vaccines, concluding that the vaccines are safe, except when the patient has very low or extremely low T cells (Table 2) (119, 120, 121, 122, 123). One study utilized a CD8 count of <200 cells/µl to define a high-risk group. Among this “high-risk” group, adverse events were mild and no more common than in the group with higher CD8 T cells (123). A larger cohort study found that patients with adverse events related to the varicella vaccine had lower CD4 percentages (24.8%) than those who did not (35.5%) (120). Yet, multiple studies noted that unvaccinated 22q11.2DS children had high rates of varicella infections. Summarizing these findings, vaccinations with live viral vaccines are safe for the vast majority of 22q11.2DS patients, the exception being those with confirmed congenital athymia (failed two TREC newborn screens and CD3^+^ T cells <50 cells/µl) (14, 40). Published vaccine guidelines should be adhered to as cases of severe disease related to live viral vaccines have been reported (124). In addition to safety issues, there have been some concerns regarding diminished antibody responses to vaccines in the 22q11.2DS cohort. The studies have been inconsistent and could be related to the age of the study population (125). The next section describes the larger landscape of humoral dysfunction.

### Humoral dysfunction

A surprising finding was that some 22q11.2DS patients were antibody deficient (Table 1) (126). Early on, low IgM was identified, but this was of unclear relevance due to recurrent infections (127). A multinational study found that 6% of patients with 22q11.2DS had hypogammaglobulinemia, and a recent United States Immunodeficiency Network (USIDNET) study revealed that 6% were on immunoglobulin therapy (128, 129). Marginal zone B cells and natural antibodies (i.e., those involved in T cell–independent responses) were lower in patients over 2 years of age (130). There is little information regarding the association of impaired antibody production and infections in 22q11.2DS patients, but in studies of patients with other immunodeficiency disorders, lower antibody levels do correlate with increased numbers of infections (131, 132). In one study, infections correlated with lower IgG levels with no association with T cell counts, while associations with TREC counts were not investigated (103). Additionally, a key insight was that poor responses to vaccines were associated with autoimmunity (126).

The humoral dysfunction, defined by poor vaccine responses or low immunoglobulin levels, is found in a relatively small subset of 22q11.2DS patients (125, 133). Yet, there is evidence in a much larger percentage of patients that the B cell compartment is altered. The κ-deleting element recombination circle is normal in childhood, suggesting that the production of B cells is normal (100, 134). Yet, the population of switched memory B cells is low in about two thirds of the older 22q11.2DS children and adults (104, 135). Patients with low switched memory B cells have a higher rate of autoimmune cytopenias (136). This is specific for autoimmune cytopenias as it was not seen for autoimmune thyroid disease or juvenile arthritis (136). The B cells have diminished somatic hypermutations compared to controls in both adults and children (137), suggesting that Tfh help is compromised despite increased numbers.

### Autoimmunity

22q11.2DS patients have a higher incidence of autoimmune disorders, the most common being thyroiditis, arthritis, and autoimmune cytopenias (108, 138, 139). Autoimmune disease has not been specifically delineated in adults; however, psoriasis and autoimmune thyroid disease appear common (111). Additional autoimmune and inflammatory diseases reported include lupus, uveitis, inflammatory bowel disease, granulomatous interstitial lung disease, and diabetes. Thus, overall autoimmunity is increased in the 22q11.2DS cohort. Studies of biomarkers or clinical characteristics that would predict the development of autoimmune disease have been undertaken by multiple groups. The most robust study of autoimmunity in 22q11.2DS were low switched memory B cells, low CD4 naïve T cells predicting autoimmune cytopenias (136). Low T cells have been recognized in patients with active autoimmunity since 2002 (126). Low overall B cells in two studies were associated with autoimmunity (139, 140) and studies have made the important observation that a history of significant infections was associated with autoimmune disease (108, 126, 141). This aligns with the observation that infection history is a risk factor for inflammation in chronic granulomatous disease (142) and it is worth considering whether that phenomenon is globally the case across most IEIs. Concordant with the idea of an altered B cell compartment in 22q11.2DS are findings, in several studies, that immunoglobulin dysfunction was associated with autoimmunity and infection (140, 141).

### Allergies

Allergies are also recognized as a common complication in 22q11.2DS. Several studies have documented increased allergies ranging from asthma to food and drug allergies (104, 140, 143). Increased Th2 cells have also been noted (144). The mechanism may be homeostatic proliferation, as is the case in Omenn syndrome (83, 104). In a cross-sectional multicenter study, allergies were associated with recurrent infections and low T cells (140). One study specifically identified low CD8 T cells in children with 22q11.2DS and allergies (108). In one study, 32% of patients had low IgM levels and this was associated with an odds ratio of 3.7 for allergies (104). Thus, the biomarkers for allergy are diverse, but the allergies appear to be enriched in those with the most disordered immune system.

### Other clinical features of 22q11.2DS

A correlative study examined the co-occurrence of various clinical phenotypes in 22q11.2DS. This study as well as others found no association of thymic hypoplasia and cardiac anomaly, hypoparathyroidism, or other clinical features (103). However, psychosis was found to be associated with clinical autoimmunity (145). In the general population, inflammation has been epidemiologically associated with schizophrenia and in the case of 22q11.2DS, both IL-6 and IL-17 have been found elevated in those patients with psychoses (146, 147).

Management of adults with 22q11.2DS has evolved as more children have survived cardiac surgery and grown to adulthood. Nevertheless, information on adults is limited. An ongoing risk of sudden cardiac death has been documented, and rates of psychosis and other mental health issues have received appropriate focus (148). A consensus guideline on management of adults has recently been developed (1).

### The added complexities of 22q11.2DS on immunity

The cytoband 22q11.2 is a complex genetic region, with the 8 highly homologous low copy repeats (LCRs) responsible for the chromosomal deletions on 22q11.2DS. These LCRs are only present in higher order primates, with humans having expanded their number to eight (LCR A–H), with LCR A existing as eight allelic variants in the population (16, 17). These LCRs may function as chromatin assembly hubs, regulating gene expression both within the 22q11.2 locus and, as recently described, >300 genes from distinct chromosomal locations (149, 150). This epigenetic regulation likely accounts for some of the clinical variability among 22q11.2DS patients, with transcriptomic differences reported in T, B, and mast cells (4, 52, 149, 151, 152). For example, RNA sequencing of circulating T cells from 22q11.2DS patients compared to controls suggested defective cell and behavior pathways along with liver X receptor/retinoid X receptor regulated processes (91). RNA sequencing of peripheral blood comparing 22q11.2DS and controls also revealed an altered gene expression in B cells and mast cells, which could result from epigenetic changes in genes outside the 22q11.2DS locus (153).

Of the genes within the 22q11.2DS locus, the haploinsufficiency of several may impact the phenotypes (Table 3). Examples include Claudin 5, a tight junction protein encoded on chr. 22q11.2 (15, 154, 155). In mouse models, a deficiency of Claudin 5 reduces T cell egress by disrupting the thymic cortex–blood barrier along with an impact on NCC-derived perivascular cell–endothelial cell interactions (156, 157). While haploinsufficiency is not a knockout, reduced levels of Claudin 5 could contribute to disrupted perivascular–endothelial functions. For example, blood vessel organoids, formed with induced pluripotent stem cells prepared from 22q11.2DS patients, are smaller than controls (158, *Preprint*). There is an increased spacing between the perivascular and endothelial cells along with more collagen and fibronectin evident in such “22q11.2” organoids (158, *Preprint*). 22q11.2DS patients have increased vascular leakage, assessed in the blood–brain barrier (154). This leakage, perhaps partly impacted by the haploinsufficiency of Claudin 5, could increase immune cell trafficking to the brain. TBX1, the key driver of the congenital malformations in 22q11.2DS, regulates vascular formation in the developing brain (159). In the mouse models of 22q11.2DS, embryonic thymuses had diminished vascularization (67, 72). Recent findings indicate a postnatal role for *TBX1* re-expression in supporting lymphangiogenesis in the heart following ischemia (159). All these findings suggest the vascular changes due to 22q11.2DS are potentially impacting either immune cell trafficking to the sites of infection and/or cell–cell interactions in the secondary lymphoid organs. Interestingly, some of the behavioral issues in mice haploinsufficient in Tbx1 improved following vitamin B12 administration (160). *CRKL* is another gene encoded on chr. 22q11.2, haploinsufficient in patients with the 3-Mb but not 1.5-Mb deletion (Fig. 1 B). Complete knockouts of *CRKL* in mice, which is prenatal lethal, lead to overlapping congenital phenotypes as for those with 22q11.2DS (161). For some years, it was thought that *CRKL* was the main driver of the human 22q11.2DS phenotype (162). *CRKL* knockdown has been shown to impact T cell function and its haploinsufficiency in 22q11.2 could affect both T and natural killer (NK) cell activities in patients. For T cells, this is not readily evident in 22q11.2DS patients, with CRKL suggested more important for NK cell functions (163, 164). Over time, *CRKL* has been felt to have more of an impact on urogenital aspects of the phenotype in 22q11.2DS (165). Glycoprotein 1b beta (*GP1bb*), also haploinsufficient due to 22q11.2DS, is involved in platelet adhesion and hemostasis. While this could explain the thrombocytopenias and increased bleeding noted in 22q11.2DS patients, thrombocytopenia is not consistently seen in all individuals (166). Again, epigenetic regulation and/or better stratification of affected individuals could reveal additional causes of the thrombocytopenia. The deletion of *Hira* or *Dgcr8* in mice (both encoded on chr. 22q11.2DS) leads to impaired hematopoiesis and compromised stem cell proliferation (167, 168). DGCR8 is a miRNA processing enzyme and its haploinsufficiency reduces the expression of diverse miRNAs (169, 170, 171, 172). miRNAs are small noncoding RNAs, 21–23 nucleotides in length, that have key roles in regulating global stress responses (173). Notably, 22q11.2DS patients, haploinsufficient in DGCR8, have a dysregulation in miRNA expression patterns, suggesting an impact on immune functions (169, 174). Perhaps due to other epigenetic changes, miRNA expression patterns can be quite hypervariable in the 22q11.2DS cohort relative to controls (174). There are several miRNAs haploinsufficient due to their being encoded on chr. 22q11.2, including miR-185. In immune cells, miR-185 targets several key transcripts involved in B and T cell receptor signaling (175). Among the targets are Bruton’s tyrosine kinase (B cells), with reduced levels of miR-185 correlating with B cell autoantibody production (176). Other miR-185 targets in immune cells include MZB1, NFATc3, and CAMK4, which are involved in antigen receptor–mediated signaling (175). In hippocampal neurons, miR-185 targets sarcoplasmic/endoplasmic reticulum calcium ATPase 2 (SERCA2) (151). In mouse models, reduced expression of miR-185 leads to presynaptic neurotransmitter release. Lastly, mitochondria appear to be dysfunctional in 22q11.2DS, a feature that appears to be of particular importance in neural stem cells (15, 177, 178). Not yet fully understood are the impacts of many lncRNAs and several sncRNAs (15–50 nucleotides in length) haploinsufficient in the 22q11.2DS cohort (4, 179, 180). Several of these are listed in Table 3, with ongoing studies addressing their contributions to 22q11.2DS. In summary, 22q11.2DS remains a complex syndrome due to both genetic and epigenetic changes that can vary from individual to individual (4).

**Table 3.** Key genetic and epigenetic modifiers encoded on chr. 22q11.2

Gene name (protein name if coding)	Gene function	Mechanism of action	Clinical phenotypes	Comparative mouse models^a^
*TBX1* (T-box transcription factor 1)	Transcription factor	Binds DNA sequences and associates with histone methyltransferases to activate transcription	Multiple and variable congenital defects (see Table 1)	KO^b^ is embryonic lethal Haploinsufficiency: Similar phenotypes as humans but much less penetrant thymic hypoplasia
*CRKL* (CRK-like)	SH2 and SH3 domain containing intracellular signaling adapter protein	Promotes intracellular signal transduction	Genetic modifier of 22q11.2DS	KO is prenatal lethal with heart, liver, and placental defects Crkl^+/−^Tbx1^+/−^ mice: CHD^c^ and thymic hypoplasia
*CLDN 5* (CLAUDIN 5)	Tight junction protein	Blood–brain barrier (BBB) integrity Cortical thymus–blood barrier	Increased vascular permeability and weakened BBB	Mouse KO leads to nonviable offspring due to defective BBB
*DGCR6* (DGCR6)	Nuclear phosphoprotein	Expressed in neural crest cells Homology to laminin-g1 chain	CHD with deletion or duplication	Haplosufficiency associated with learning deficit
*DGCR8* (DGCR8)	MicroRNA processing enzyme	Required for miRNA biogenesis	Processing microRNAs in immune cells and neural progenitors	KO is embryonic lethal at E6.5
MiR-185	MicroRNA	Targets SERCA2, BTK, MZB1, NFAT, CAM4K	Potential contributor to autoantibody production	KO is normal: Increased bone formation during osteogenesis Haploinsufficiency correlated with increased autoantibody
*DGCR5*	LncRNA	Regulator of alternative splicing	Unknown	No mouse model

a Phenotypic differences exist in strains used.

b Knockout.

c Congenital Heart Disease

## Summary

22q11.2DS is a complex syndrome arising during embryogenesis that impacts many organ systems. The impacts of the deletion on immune function are similarly diverse, with direct effects related to thymic hypoplasia and indirect consequences resulting from altered thymic and/or secondary lymphoid environments. While the driver of these effects on the immune system is primarily related to the severity of T cell lymphopenia, cell types including innate lymphoid cells, B cells, NK cells, and mast cells may also be affected.
